# Analysis of the geometric phase for a nanowire-bridged superconducting Fabry-Perot resonator

**DOI:** 10.1038/s41598-019-44754-7

**Published:** 2019-06-10

**Authors:** Jeong Ryeol Choi, Sanghyun Ju

**Affiliations:** Department of Physics, Kyonggi University, Yeongtong-gu, Suwon, Gyeonggi-do 16227 Republic of Korea

**Keywords:** Theoretical physics, Quantum optics, Computational methods, Quantum mechanics

## Abstract

The geometric phases of a nanowire-bridged superconducting Fabry-Perot resonator subjected to a microwave transmission have been investigated through its modelling into a RLC-circuit. Because the Hamiltonian of the system is a somewhat complicated form, special mathematical techniques, such as the invariant operator method and the unitary transformation approach, have been adopted in order to treat the system; These methods are very useful for managing complicated time-dependent Hamiltonian systems. We have rigorously evaluated the analytical geometric phases in both the Fock and coherent states. Typically, the geometric phases oscillate and the amplitude of such oscillations tend to grow over time. The influence of parameters of the system on the geometric phases has been analyzed in detail through the relevant illustrations. From our research, the concept of geometric phases and associated quantum mechanical characters of the system has been clarified. Our investigation for the geometric phases is useful for understanding topological features of the system, that take place through the evolution of the wave functions.

## Introduction

Nanowires became a principal research subject in the science community thanks to their potential applicability in broad areas of modern technology, such as the nanowire-bridged resonators^1,2^, AC power source generations^3,4^, and nanowire cantilevers^5^. We will focus on the nanowire-bridged superconducting Fabry-Perot resonators in this work, since they can be used as building blocks for superconducting qubits in quantum computations. The possibility of qubit fabrications with nanowire-resonator systems stems from the fact that we can control them to exhibit the properties of many-body cavity QEDs under appropriate situations. After the first experimental report in this line by Manucharyan *et al*.^1^, active subsequent researches have been followed^2,6–12^. According to this trend, it may be highly required to carry out the research that deepens the understanding of their underlying physical mechanisms.

In phenomenological models for describing the amplitude of the field-driven charge oscillations in resonator-nanowire systems, quantal wave phenomena with a phase evolution appear in general. We considered this and took into attention for the evolution of waves including their phases inside the wires in order to elucidate detailed theoretical features of the phenomena. In view of gauge symmetry theory, there are broad implications associated to geometric phases of the waves that appear in diverse nanodevices^13–16^.

Although Herzberg and Longuet-Higgins^17^ recognized a signature of a geometric phase evolution in the Born-Oppenheimer electronic wave functions in 1963, it had not actively been recognized for a long time in physics until the advent of Berry’s seminal discovery^18^. Berry reported^18^ that there are additional phase accumulations (now known as the geometric phase) for a time-varying system during its wave evolution through a closed path in the parameter space. Berry’s discovery had triggered an active research on phase dynamics in adiabatic quantum systems including nanodevices. The subsequent researches on this issue had remarkably contributed to the development of physics associated with topology and its generalizations, meanwhile widespread interest for the geometric phase continues to this day. The geometric phase is a parallel transport or a holonomy that can be represented in terms of unit vectors in the Hilbert space.

To attain theoretical insights for dynamics of the nanowire-bridged superconducting Fabry-Perot resonators, we may need to investigate the geometric phase. The applications of geometric phases in characterising the properties of nanowires are ubiquitous: They include observations of conductance fluctuations through quantum Hall effects^19^, estimations of piezoelectricity in semiconductors^20–23^, effects of ambipolar fields in crystalline insulators^24^, and the applications as a tool to study many-body quantum systems in and out equilibrium conditions^25–27^.

A geometric phase is defined as the quantal phase of geometric origin included in the wave function during a fixed time that corresponds to one-period evolution of the eigenstate through a closed circuit^28,29^. We can also define the geometric phase in a slightly different manner, where we consider the geometric phase accumulated during an arbitrary time *t* instead of one-period or one-cycle (see, for example, Eq. (4.23) in Ref. 30). While the geometric phase in the former definition gives its well defined quantity for a specific system, the geometric phase in the latter is usually represented in terms of *t*. We will adopt the latter definition in this work, because that definition gives a richer relation between a time-dependent Hamiltonian system and its quantal-phase properties, which admits a time-dependent change of the geometric phase. For more general discussions on geometric phases and their effects, refer to Ref. 31.

We will investigate the geometric phases of the system in the Fock and the coherent states which are fundamental quantum states in physics. In particular, a coherent state is a classical-like state that can be used to demonstrate the correspondence between quantum and classical states. We will quantitatively describe the mechanics of the system, by modelling it as a lumped single-series RLC circuit around the resonance frequency. Useful methods for treating time-dependent Hamiltonians, such as invariant operator methods^32–34^ and unitary transformation methods^34^, will be adopted in order to facilitate the analytical analysis of the system through quantum mechanical points of view. To understand the theoretical mechanisms for the nanowire-bridged resonators, which are somewhat unclear now while the knowledge about them is necessary in implementing the system as a resource of quantum computation, an investigation of the geometric phase may be helpful.

## Results and Discussion

### Hamiltonian and invariant operator

Nanowires can be made to act as dissipationless inductors, and this fact was experimentally demonstrated by Ku *et al*. through the observation of kinetic inductance in the nanowire connected to a superconducting coplanar waveguide resonator^1^. In actual systems, the superconducting Fabry-Perot resonators are interrupted by a gap at its center and a superconducting nanowire has been suspended across the gap as a bridge. There is an excitation of the plasma oscillation in the resonator by an input microwave signal, leading to the occurrence of a supercurrent that flows through the nanowire. For a more detailed experimental setup of the system, refer to Refs. 1 and 2.

As a preliminary step for investigating the geometric phases, we first need to setup an equation of motion that governs the behavior of the system. Then, it is necessary to derive quantum states in which we develop the geometric phases from the Schrödinger equation. We are interested in the geometric phases in the Fock and the coherent states in this work. For the time-dependent Hamiltonian systems (TDHSs), such states can be derived from the invariant operator method together with the unitary transformation approach.

From the model of resonators that have a nanowire as an inductive element, it is possible to describe them as equivalent lumped single-series RLC electronic circuits at the frequency near the resonance, allowing us to investigate the amplitudes of the oscillating charge quantitatively and the supercurrent signal for the system driven by a voltage supply. In this situation, the nonlinear equation for the charge *q* stored on the capacitor in the effective circuit of the nanowire-bridged resonator is given by^1,35^

1 $$({L}_{{\rm{eff}}}+\frac{{L}_{{\rm{J}}}}{\sqrt{1-{\dot{q}}^{2}/{I}_{0}^{2}}})\ddot{q}+{R}_{{\rm{eff}}}\dot{q}+\frac{1}{{C}_{{\rm{eff}}}}q+{V}_{{\rm{NW}}}={V}_{{\rm{eff}}}\,\cos (\omega t+\varphi ),$$

where *V*_NW_ is the voltage between the ends of the nanowire, *R*_eff_, *L*_eff_, and *C*_eff_ are the resistance, inductance, and capacitance in the circuit effectively representing the resonator, respectively, and *V*_eff_ is amplitude of the effective voltage of the driving source. By using the approximation ${(1-{\dot{q}}^{2}/{I}_{0}^{2})}^{-\mathrm{1/2}}=1+{\dot{q}}^{2}/(2{I}_{0}^{2})$ in the weak non-linear limit where $|\dot{q}|\ll {I}_{0}$^1,35^, we have the Duffing oscillator equation of the form

2 $${L}_{{\rm{T}}}(1+\frac{{L}_{{\rm{J}}}}{{L}_{{\rm{T}}}}\frac{{\dot{q}}^{2}}{2{I}_{0}^{2}})\ddot{q}+{R}_{{\rm{eff}}}\dot{q}+\frac{1}{{C}_{{\rm{eff}}}}q=-\,{V}_{{\rm{NW}}}+{V}_{{\rm{eff}}}\,\cos (\omega t+\varphi ),$$

where *L*_T_ = *L*_eff_ + *L*_J_. Because *L*_J_ ≤ *L*_T_, this equation can be further simplified under the same weak non-linear limit as

3 $${L}_{{\rm{T}}}\ddot{q}+{R}_{{\rm{eff}}}\dot{q}+\frac{1}{{C}_{{\rm{eff}}}}q=-\,{V}_{{\rm{NW}}}[1-\kappa \,\cos (\omega t+\varphi )],$$

where *κ* = *V*_eff_/*V*_NW_.

This system can be described by the Caldirola-Kanai Hamiltonian that is given, in view of quantum mechanics, by

4 $$\hat{H}={e}^{-\beta t}\frac{{\hat{p}}^{2}}{2{L}_{{\rm{T}}}}+\frac{1}{2}{e}^{\beta t}\{{L}_{{\rm{T}}}{\omega }_{0}^{2}{\hat{q}}^{2}+2{V}_{{\rm{NW}}}\mathrm{[1}-\kappa \,\cos (\omega t+\varphi )]\hat{q}\},$$

where $\hat{p}=-\,i\hslash \partial /\partial q$, *β* = *R*_eff_/*L*_T_, and ${\omega }_{0}=1/\sqrt{{L}_{{\rm{T}}}{C}_{{\rm{eff}}}}$. It may be very difficult to derive quantum solutions of the system on the basis of the separation of variables method, because this Hamiltonian is a somewhat complicated time-dependent form driven by a time-varying force. Notice that the time functions in Eq. (4) cannot be easily separated out from canonical variables. For this reason, we may need to adopt other potential mathematical tools for treating the system quantum mechanical points of view. In this case, the invariant operator method^32,33^ is useful because the eigenstates of the invariant operator are in the kernel of the quantum dynamical problem. Hence, we introduce an invariant operator of the system from the Hamiltonian given in Eq. (4). Using the Liouville-von Neumann equation for an invariant $\hat{ {\mathcal I} }$, which is given by $d\hat{ {\mathcal I} }/dt=\partial \hat{ {\mathcal I} }/\partial t+[\hat{ {\mathcal I} },\hat{H}]/(i\hslash )=0$, we derive a linear invariant operator as^36^

5 $$\hat{ {\mathcal I} }=\hat{A}{e}^{i{\rm{\Omega }}t},$$

where $\hat{a}$ is an annihilation operator of the system and ${\rm{\Omega }}=\sqrt{{\omega }_{0}^{2}-{\beta }^{2}\mathrm{/4}}$: The exact formula of $\hat{A}$ is given by

6 $$\hat{A}=C[{L}_{{\rm{T}}}({\rm{\Omega }}+i\frac{\beta }{2}){e}^{\beta t\mathrm{/2}}[\hat{q}-{Q}_{p}(t)]+i{e}^{-\beta t\mathrm{/2}}[\hat{p}-{P}_{p}(t)]],$$

where *C* = (2*ℏL*_T_Ω)^−1/2^, and *Q*_*p*_(*t*) and *P*_*p*_(*t*) are particular solutions of the classical equation of motion of the system in *q* and *p* spaces, respectively. From Eq. (4), we derive

7 $${Q}_{p}(t)={K}_{1}\,\cos (\omega t+\varphi -\delta )-{K}_{2},$$

where $${K}_{1}=K\kappa /\sqrt{{({\omega }_{0}^{2}-{\omega }^{2})}^{2}+{\beta }^{2}{\omega }^{2}}$$, $${K}_{2}=K/{\omega }_{0}^{2}$$, *K* = *V*_NW_/*L*_T_, and *δ* is a phase of the form $$\delta ={\tan }^{-1}[\beta \omega /$$$$({\omega }_{0}^{2}-{\omega }^{2})],$$ while

8 $${P}_{p}(t)=-\,{\bar{K}}_{1}{e}^{\beta t}\,\sin (\omega t+\varphi -\delta ),$$

where ${\bar{K}}_{1}={L}_{{\rm{T}}}\omega {K}_{1}$. We can check that $\hat{ {\mathcal I} }$ is a quantum invariant quantity by demonstrating that the direct differentiation of Eq. (5) with respect to time results in zero. Notice that the Hermitian adjoint of Eq. (5), ${\hat{ {\mathcal I} }}^{\dagger }$, which is described by the creation operator ${\hat{A}}^{\dagger }$, is also an invariant operator. By a straightforward evaluation, we can easily confirm that $[\hat{A},{\hat{A}}^{\dagger }]=1$.

### Unitary transformation

If we write the eigenvalue equation of $\hat{A}$ as $\hat{A}|A\rangle =A|A\rangle$, |*A*〉 is the coherent state of the system. We will investigate the geometric phase of the system in the coherent state, as well as in the Fock state, with the help of the invariant operator. Because the original invariant operator given in Eq. (5) is a somewhat complicated form, it is favorable to manage the invariant operator after transforming it into a simple form by means of the unitary transformation method. For this reason, we introduce a unitary operator $\hat{U}={\hat{U}}_{1}{\hat{U}}_{2}{\hat{U}}_{3}$, where

9 $${\hat{U}}_{1}=\exp (i{P}_{p}(t)\hat{q}/\hslash )\exp (\,-\,i{Q}_{p}(t)\hat{p}/\hslash ),$$

10 $${\hat{U}}_{2}=\exp [i\beta t(\hat{q}\hat{p}+\hat{p}\hat{q}\mathrm{)/(4}\hslash )],$$

11 $${\hat{U}}_{3}=\exp [\,-\,i{R}_{{\rm{eff}}}{\hat{q}}^{2}\mathrm{/(4}\hslash \mathrm{)].}$$

From the unitary transformation $\hat{I}={\hat{U}}^{-1}\hat{ {\mathcal I} }\hat{U}$, we have

12 $$\hat{I}=\hat{a}{e}^{i{\rm{\Omega }}t},$$

where $\hat{a}$ is the annihilation operator of the simple harmonic oscillator (SHO), that is given by

13 $$\hat{a}=\sqrt{\frac{{L}_{{\rm{T}}}{\rm{\Omega }}}{2\hslash }}\hat{q}+\frac{i\hat{p}}{\sqrt{2{L}_{{\rm{T}}}{\rm{\Omega }}\hslash }}.$$

By using a fundamental dynamics based on the Hamiltonian, it can be proved that

14 $$\hat{a}(t)=\hat{a}\mathrm{(0)}{e}^{-i{\rm{\Omega }}t}\mathrm{.}$$

We can confirm from this consequence that the transformed invariant operator is represented in terms of the SHO annihilation operator. Hence, the transformed system just corresponds to the SHO of which the classical and quantum solutions are well known. The general classical solutions for the canonical variables in the transformed system are thus given by the SHO solutions:

15 $${q}_{{\rm{cl}}}={q}_{0}\,\cos ({\rm{\Omega }}t+\phi ),$$

16 $${p}_{{\rm{cl}}}=-\,{L}_{{\rm{T}}}{\rm{\Omega }}{q}_{0}\,\sin ({\rm{\Omega }}t+\phi \mathrm{).}$$

The wave functions in the transformed system in the Fock state are expressed in the forms

17 $$\langle q|{\varphi }_{n}\rangle ={(\frac{\sqrt{\varepsilon /\pi }}{{2}^{n}n!})}^{\mathrm{1/2}}{H}_{n}(\sqrt{\varepsilon }q){e}^{-\varepsilon {q}^{2}\mathrm{/2}},$$

where *ε* = *L*_T_Ω/*ℏ*, which also correspond to the SHO wave functions.

The eigenvalue equation of the annihilation operator in the transformed system can be written as

18 $$\hat{a}|\alpha \rangle =\alpha |\alpha \rangle \mathrm{.}$$

The eigenvalue of the above equation is given by

19 $$\alpha (t)=\alpha \mathrm{(0)}{e}^{-i{\rm{\Omega }}t},$$

where *α*(0) = *α*_0_*e*^−*iφ*^ with ${\alpha }_{0}=\sqrt{{L}_{{\rm{T}}}{\rm{\Omega }}\mathrm{/(2}\hslash )}{q}_{0}$.

On the other hand, the eigenstate can be written as^37^

20 $$\langle q|\alpha \rangle ={(\frac{{L}_{{\rm{T}}}{\rm{\Omega }}}{\hslash \pi })}^{\mathrm{1/4}}\exp (-\frac{{L}_{{\rm{T}}}{\rm{\Omega }}}{2\hslash }{q}^{2}+\sqrt{\frac{2{L}_{{\rm{T}}}{\rm{\Omega }}}{\hslash }}\alpha q-\frac{|\alpha {|}^{2}}{2}-\frac{{\alpha }^{2}}{2}).$$

This is the normalized wave function in the coherent state. The quantum dynamics of the system developed here will be used in the subsequent subsections in order to derive geometric phases and to analyze them.

### Geometric phases in the Fock state

We first investigate the geometric phases in the Fock state which is the most elementary quantum state. For convenience, we will call the wave functions in which the phases have been removed as the “eigenfunctions” while we denote them in the forms |Φ_*n*_(*t*)〉 in the Fock state. Then, the wave functions in the Fock state can be represented to be |Ψ_*n*_(*t*)〉 = |Φ_*n*_(*t*)〉exp[*iγ*_*n*_(*t*)] where *γ*_*n*_(*t*) are overall phases. Not only the phases but the eigenfunctions as well are expressed in terms of *t* for TDHSs. However, when the time-dependence of the Hamiltonian has been removed and thereby the system has become the SHO, the time-dependence of the eigenfunction in the Fock state also disappears.

The geometric phases are represented in terms of the eigenfunctions such that

21 $${\gamma }_{G,n}(t)={\int }_{0}^{t}\langle {{\rm{\Phi }}}_{n}(t^{\prime} )|i\frac{\partial }{\partial t^{\prime} }|{{\rm{\Phi }}}_{n}(t^{\prime} )\rangle dt^{\prime} +{\gamma }_{G,n}\mathrm{(0).}$$

Using the unitary transformation relation $|{{\rm{\Phi }}}_{n}\rangle =\hat{U}|{\varphi }_{n}\rangle$, we can express them as

22 $${\gamma }_{G,n}(t)={\int }_{0}^{t}\langle {\varphi }_{n}|{\hat{U}}^{-1}i\frac{\partial }{\partial t^{\prime} }\hat{U}|{\varphi }_{n}\rangle dt^{\prime} +{\gamma }_{G,n}\mathrm{(0).}$$

From a minor evaluation using Eqs. (9)–(11), they become

23 $${\gamma }_{G,n}(t)={\int }_{0}^{t}\langle {\varphi }_{n}|{\textstyle (}\hat{X}(\hat{q},\hat{p},t^{\prime} )+i\frac{{\rm{\partial }}}{{\rm{\partial }}t^{\prime} }{\textstyle )}|{\varphi }_{n}\rangle dt^{\prime} +{\gamma }_{G,n}(0),$$

where^38^

24 $$\begin{array}{ccc}\hat{X}(\hat{q},\hat{p},t) & = & -\frac{1}{\hslash }{\textstyle [}{\dot{P}}_{p}(t)[\hat{q}{e}^{-\beta t/2}+{Q}_{p}(t)]\\  &  & -{\dot{Q}}_{p}(t){e}^{\beta t/2}{\textstyle (}\hat{p}-\frac{{R}_{{\rm{e}}{\rm{f}}{\rm{f}}}}{2}\hat{q}{\textstyle )}+\frac{\beta }{4}(\hat{q}\hat{p}+\hat{p}\hat{q}-{R}_{{\rm{e}}{\rm{f}}{\rm{f}}}{\hat{q}}^{2}){\textstyle ]}.\end{array}$$

**Figure 1:**
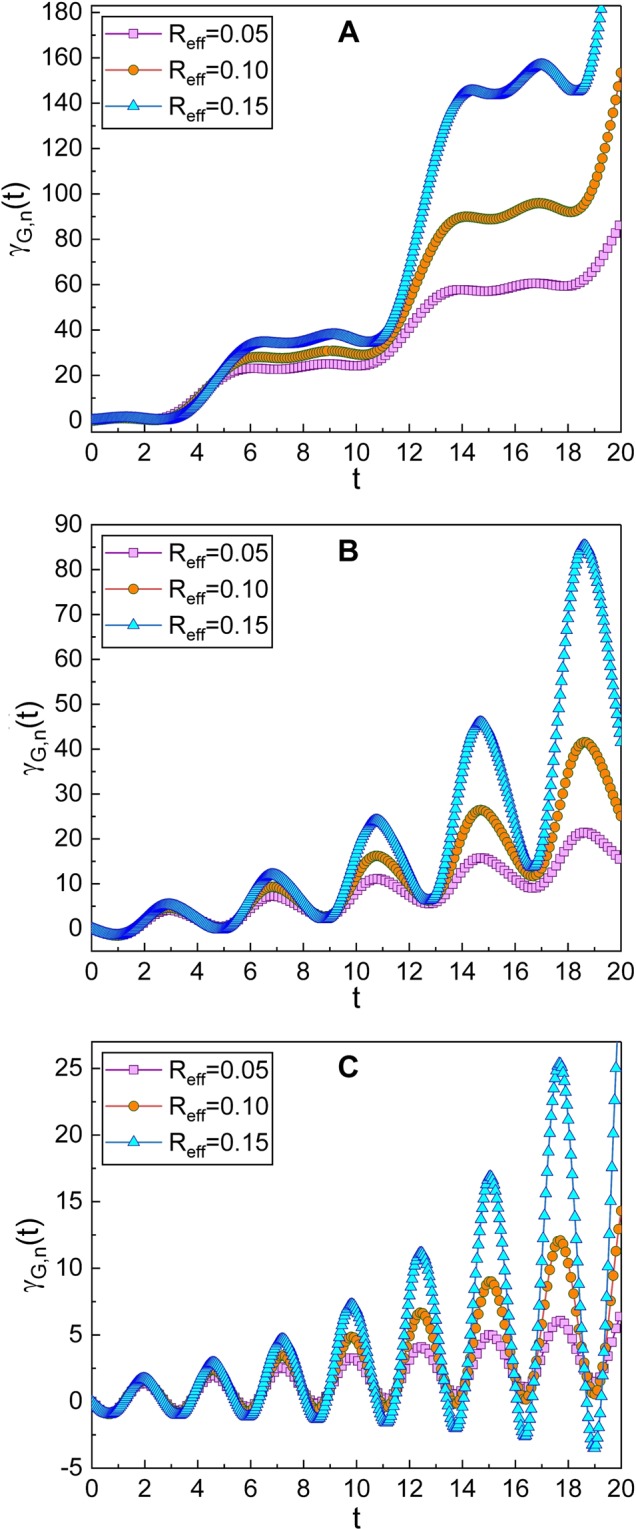
Time evolution of the geometric phase in the Fock state for several values of *R*_eff_. We have used *ω* = 0.8 for (**A**), *ω* = 1.6 for (**B**) and *ω* = 2.4 for (**C**). We have chosen other parameters as *L*_T_ = 1, *C*_eff_ = 1, *V*_NW_ = 2, *V*_eff_ = 1, *n* = 0, *ℏ* = 1, *γ*_G,*n*_(0) = 0, and *ϕ* = *φ* = 0.

Further computation in the configuration space using Eq. (17) results in

25 $${\gamma }_{G,n}(t)=\frac{{\beta }^{2}t}{4{\rm{\Omega }}}(n+\frac{1}{2})+\frac{{\bar{K}}_{1}}{\hslash }\eta (t)+{\gamma }_{G,n}\mathrm{(0),}$$

where *η*(*t*) is a time function: The exact formula of *η*(*t*) is

26 $$\eta (t)={K}_{1}[\beta {f}_{1}(t)+\omega {f}_{2}(t)]-{K}_{2}[\beta {f}_{3}(t)+\omega {f}_{4}(t)],$$

where *f*_*i*_(*t*) (*i* = 1, 2, 3, 4) are given by (see METHODS section)

27 $${f}_{i}(t)={F}_{i}(t)-{F}_{i}\mathrm{(0)},$$

with

28 $${F}_{1}(t)=\frac{-{e}^{\beta t}}{\mathrm{2(}{\beta }^{2}+4{\omega }^{2})}\{2\omega \,\cos \,[2\theta (t)]-\beta \,\sin \,[2\theta (t)]\},$$

29 $${F}_{2}(t)=\frac{{e}^{\beta t}}{2\beta ({\beta }^{2}+4{\omega }^{2})}\{4{\omega }^{2}+{\beta }^{2}\{1+\,\cos \,[2\theta (t)]\}+2\beta \omega \,\sin \,[2\theta (t)]\},$$

30 $${F}_{3}(t)=\frac{-{e}^{\beta t}}{{\beta }^{2}+{\omega }^{2}}[\omega \,\cos \,\theta (t)-\beta \,\sin \,\theta (t)],$$

31 $${F}_{4}(t)=\frac{{e}^{\beta t}}{{\beta }^{2}+{\omega }^{2}}[\omega \,\sin \,\theta (t)+\beta \,\cos \,\theta (t)],$$

while *θ*(*t*) = *ωt* + *ϕ* − *δ*. Thus, Eq. (25) with Eqs. (26)–(31) are exact analytical formulas of the geometric phases in the Fock state. The time behavior of these phases has been plotted in Fig. 1 for several values of *R*_eff_. Roughly speaking, the geometric phase oscillates and the oscillation amplitude increases over time. The amplitude of such an oscillation grows as *R*_eff_ becomes large. For the case of Fig. 1(A) that corresponds to the relatively small *ω*, the oscillatory behaviour is not very evident. However, as *ω* grows, the oscillation of the geometric phase becomes more distinct as shown in Fig. 1(B,C).

Lee *et al*.^29^ also studied geometric phases in the Fock state of a system that corresponds to a particular case of ours (for details of this, see METHODS). Because they considered a geometric-phase shift accompanying the evolution of the state round a closed circuit for only a period in the parameter space, their resulting geometric phase, which is Eq. (5.16) in Ref. 29, is independent of time; That phase does not depend on the quantum number *n* as well, meanwhile this consequence is caused by the condition *β* = 0 that they have adopted. Because Eq. (25) in the present research is irrelevant to *n* in the case where *β* → 0, our results are reconciled with those of Lee *et al*. in the same limit.

### Geometric phase in the coherent state

Now, let us investigate the geometric phase in the coherent state which resembles classical states. The geometric phase in the coherent state can be defined in terms of |*A*(*t*)〉 as

32 $${\gamma }_{G,{\rm{c}}}(t)={\int }_{0}^{t}\langle A(t^{\prime} )|i\frac{\partial }{\partial t^{\prime} }|A(t^{\prime} )\rangle dt^{\prime} +{\gamma }_{G,{\rm{c}}}\mathrm{(0).}$$

Using the relation between |*A*〉 and |*α*〉: $|A\rangle =\hat{U}|\alpha \rangle$, we have

33 $${\gamma }_{G,{\rm{c}}}(t)={\int }_{0}^{t}\langle \alpha (t^{\prime} )|{\textstyle (}\hat{X}(\hat{q},\hat{p},t^{\prime} )+i\frac{{\rm{\partial }}}{{\rm{\partial }}t^{\prime} }{\textstyle )}|\alpha (t^{\prime} )\rangle dt^{\prime} +{\gamma }_{G,{\rm{c}}}(0),$$

where $\hat{X}(\hat{q},\hat{p},t)$ is given in Eq. (24). Now we use Eq. (20) and the relation ${\int }_{-\infty }^{\infty }dq[\langle \alpha |q\rangle \partial \langle q|\alpha \rangle /\partial t]=-\,i{\rm{\Omega }}{\alpha }_{0}^{2}$. Then

34 $${\gamma }_{G,{\rm{c}}}(t)={\int }_{0}^{t}(\tilde{X}(t^{\prime} )+{\rm{\Omega }}{\alpha }_{0}^{2})dt^{\prime} +{\gamma }_{G,{\rm{c}}}\mathrm{(0),}$$

where

35 $$\begin{array}{rcl}\tilde{X}(t) & = & -\frac{1}{\hslash }[{\dot{P}}_{p}(t)(\sqrt{\frac{2\hslash }{{L}_{{\rm{T}}}{\rm{\Omega }}}}{\alpha }_{0}\,\cos ({\rm{\Omega }}t+\phi ){e}^{-\beta t\mathrm{/2}}+{Q}_{p}(t))\\  &  & +\sqrt{2\hslash {L}_{{\rm{T}}}}{e}^{\beta t\mathrm{/2}}{\alpha }_{0}{\dot{Q}}_{p}(t)(\sqrt{{\rm{\Omega }}}\,\sin ({\rm{\Omega }}t+\phi )+\frac{\beta }{2\sqrt{{\rm{\Omega }}}}\,\cos ({\rm{\Omega }}t+\phi ))\\  &  & -\frac{\hslash \beta }{4}(2{\alpha }_{0}^{2}\,\sin \,\mathrm{[2(}{\rm{\Omega }}t+\phi )]+\frac{\beta }{{\rm{\Omega }}}\mathrm{[2}{\alpha }_{0}^{2}{\cos }^{2}({\rm{\Omega }}t+\phi )+\mathrm{1/2]})].\end{array}$$

Further evaluation with the integration in Eq. (34) gives

36 $$\begin{array}{rcl}{\gamma }_{G,{\rm{c}}}(t) & = & \frac{{\beta }^{2}t}{8{\rm{\Omega }}}+{\rm{\Omega }}{\alpha }_{0}^{2}t+\frac{{\bar{K}}_{1}}{\hslash }\xi (t)+\frac{2{\alpha }_{0}^{2}\beta }{4}({f}_{7}(t)+\frac{\beta }{{\rm{\Omega }}}{f}_{8}(t))\\  &  & +\sqrt{\frac{2{L}_{{\rm{T}}}}{\hslash }}{\alpha }_{0}{K}_{1}\omega (\frac{\beta }{2\sqrt{{\rm{\Omega }}}}{f}_{5}(t)+\sqrt{{\rm{\Omega }}}{f}_{9}(t))+{\gamma }_{G,{\rm{c}}}\mathrm{(0),}\end{array}$$

where *ξ*(*t*) is another time function: The exact formula of *ξ*(*t*) is given by

37 $$\xi (t)=\eta (t)+\sqrt{\frac{2\hslash }{{L}_{{\rm{T}}}{\rm{\Omega }}}}{\alpha }_{0}[\beta {f}_{5}(t)+\omega {f}_{6}(t)],$$

where (see METHODS)

38 $${f}_{i}(t)={F}_{i}(t)-{F}_{i}\mathrm{(0})\,{\rm{for}}\,i=5,\,6,\,{\rm{and}}\,9,$$

39 $${f}_{7}(t)=\frac{1}{{\rm{\Omega }}}\,\sin ({\rm{\Omega }}t)\sin ({\rm{\Omega }}t+2\phi ),$$

40 $${f}_{8}(t)=\frac{1}{4{\rm{\Omega }}}\mathrm{\{2}{\rm{\Omega }}t-\,\sin \,\mathrm{(2}\phi )+\,\sin \,\mathrm{[2(}{\rm{\Omega }}t+\phi )]\},$$

with

41 $$\begin{array}{rcl}{F}_{5}(t) & = & -{e}^{\beta t\mathrm{/2}}(\frac{\mathrm{2(}\omega +{\rm{\Omega }})\cos \,{\theta }_{+}(t)-\beta \,\sin \,{\theta }_{+}(t)}{{\beta }^{2}+\mathrm{4(}\omega +{\rm{\Omega }}{)}^{2}}\\  &  & +\frac{\mathrm{2(}\omega -{\rm{\Omega }})\cos \,{\theta }_{-}(t)-\beta \,\sin \,{\theta }_{-}(t)}{{\beta }^{2}+\mathrm{4(}\omega -{\rm{\Omega }}{)}^{2}}),\end{array}$$

42 $$\begin{array}{rcl}{F}_{6}(t) & = & {e}^{\beta t\mathrm{/2}}(\frac{\mathrm{2(}\omega +{\rm{\Omega }})\sin \,{\theta }_{+}(t)+\beta \,\cos \,{\theta }_{+}(t)}{{\beta }^{2}+\mathrm{4(}\omega +{\rm{\Omega }}{)}^{2}}\\  &  & +\frac{\mathrm{2(}\omega -{\rm{\Omega }})\sin \,{\theta }_{-}(t)+\beta \,\cos \,{\theta }_{-}(t)}{{\beta }^{2}+\mathrm{4(}\omega -{\rm{\Omega }}{)}^{2}}),\end{array}$$

43 $$\begin{array}{rcl}{F}_{9}(t) & = & {e}^{\beta t\mathrm{/2}}(\frac{-\mathrm{2(}\omega +{\rm{\Omega }})\sin \,{\theta }_{+}(t)-\beta \,\cos \,{\theta }_{+}(t)}{{\beta }^{2}+\mathrm{4(}\omega +{\rm{\Omega }}{)}^{2}}\\  &  & +\frac{\mathrm{2(}\omega -{\rm{\Omega }})\sin \,{\theta }_{-}(t)+\beta \,\cos \,{\theta }_{-}(t)}{{\beta }^{2}+\mathrm{4(}\omega -{\rm{\Omega }}{)}^{2}}),\end{array}$$

while *θ*_±_(*t*) = *θ*(*t*) ± (Ω*t* + *φ*). Thus, we have obtained the full description of the geometric phase in the coherent state which is Eq. (36) with Eqs. (37)–(43). As you can see, this is somewhat more complicated than those of the Fock state that are given in the previous subsection. We have also plotted the time evolution of this phase in Figs. 2–4. By comparing Fig. 2 with Fig. 1, that has been drawn for several values of *R*_eff_, we can confirm that the geometric phase in the coherent state is not so much different from those of the Fock state, while the evolution of wave functions in the two states are quite different from each other. However, one can find a slight difference between the evolutions of the two classes of the geometric phases. Remember that the wave functions in the Fock state obtained from standard quantum mechanics do not oscillate with time, whereas the wave function in the coherent state oscillates as time goes by like a classical state. Figures 3 and 4 show the dependence of the geometric phase on the effective capacitance *C*_eff_ and the classical amplitude *q*_0_, respectively. As the value of *C*_eff_ increases, the amplitude of the geometric-phase oscillation also becomes large. For the large value of *q*_0_, the increment of the geometric phase over time is more rapid. The consequence of our research provides a theoretical background associated with the phases of the wave functions, which is necessary for understanding mechanical and physical properties of the system regarding topological features.

**Figure 2:**
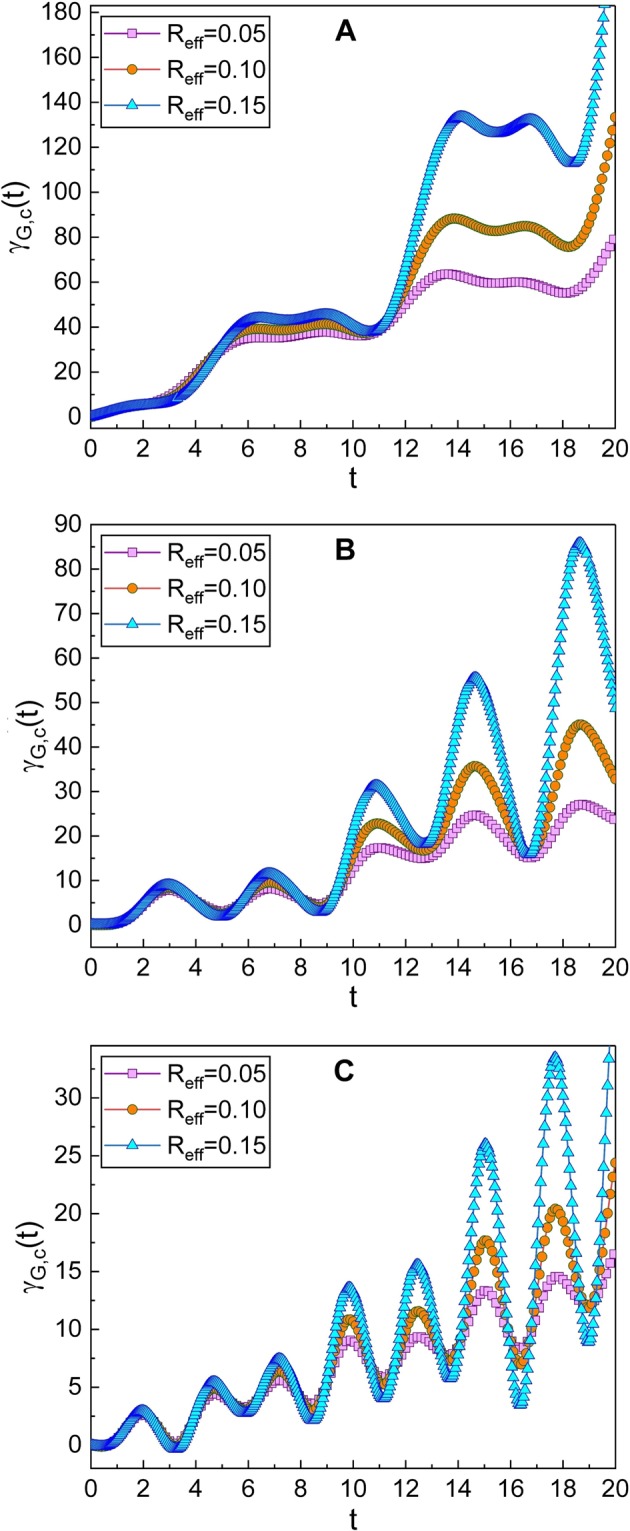
Time evolution of the geometric phase in the coherent state for several values of *R*_eff_. We have used *ω* = 0.8 for (**A**), *ω* = 1.6 for (**B**) and *ω* = 2.4 for (**C**). We have chosen other parameters as *L*_T_ = 1, *C*_eff_ = 1, *V*_NW_ = 2, *V*_eff_ = 1, *q*_0_ = 1, *ℏ* = 1, *γ*_G,*c*_(0) = 0, and *ϕ* = *φ* = 0.

**Figure 3:**
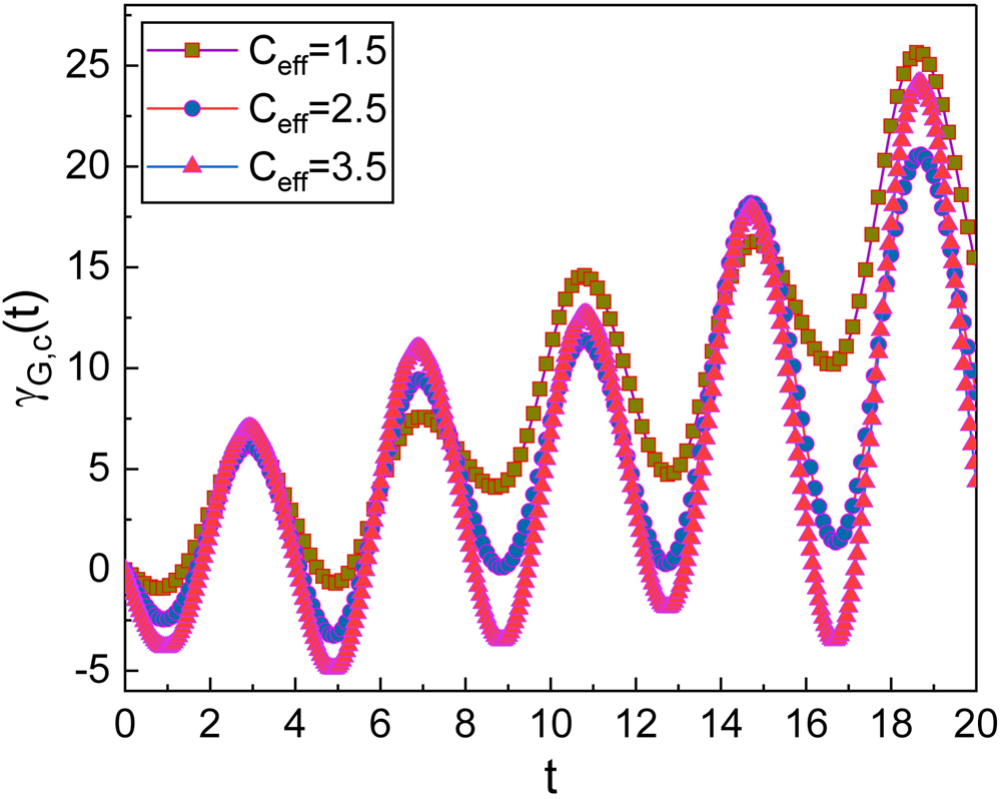
Time evolution of the geometric phase in the coherent state for several values of *C*_eff_. We have chosen the parameters as *L*_T_ = 1, *R*_eff_ = 0.05, *V*_NW_ = 2, *V*_eff_ = 1, *ω* = 1.6, *q*_0_ = 1, *ℏ* = 1, *γ*_G,*c*_(0) = 0, and *ϕ* = *φ* = 0.

**Figure 4:**
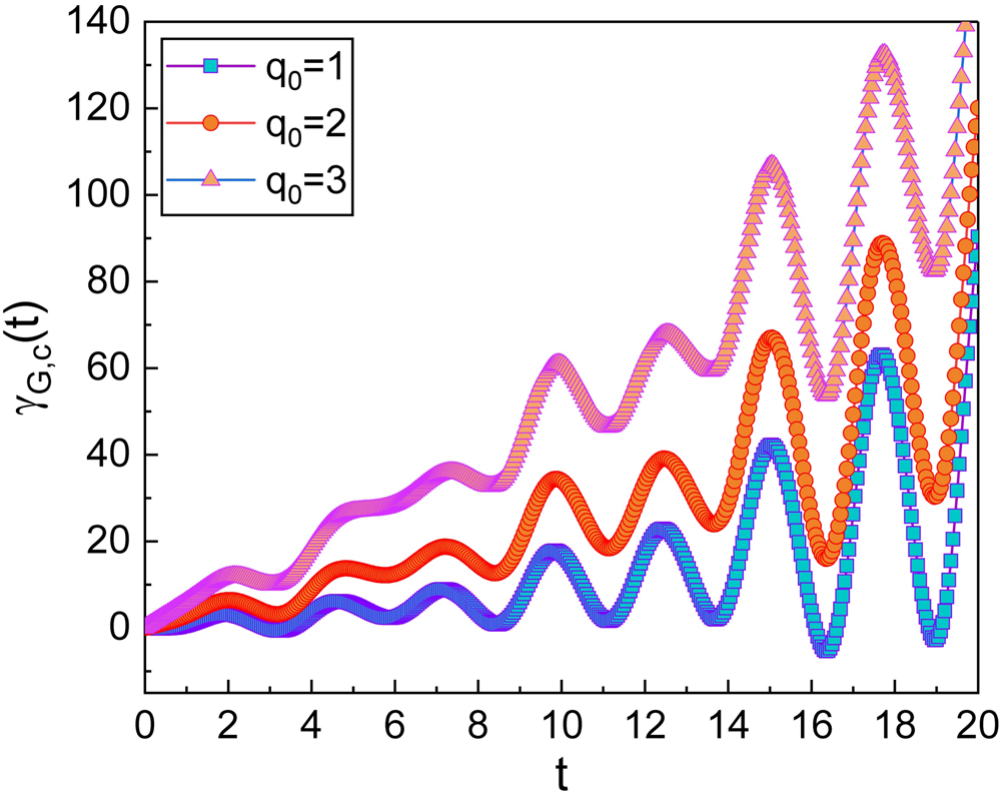
Time evolution of the geometric phase in the coherent state for several values of *q*_0_. We have chosen the parameters as *L*_T_ = 1, *C*_eff_ = 1, *R*_eff_ = 0.2, *V*_NW_ = 2, *V*_eff_ = 1, *ω* = 2.4, *ℏ* = 1, *γ*_G,*c*_(0) = 0, and *ϕ* = *φ* = 0.

### Geometric phase under some limitations and miscellaneous problems

From the previous two subsections, we have seen that the oscillations of the geometric phase become larger and larger as time goes by and with increasing damping constant *β* (or *R*_eff_). To understand the reason why these phenomena arose, let us see for some restricted cases.

**Figure 5:**
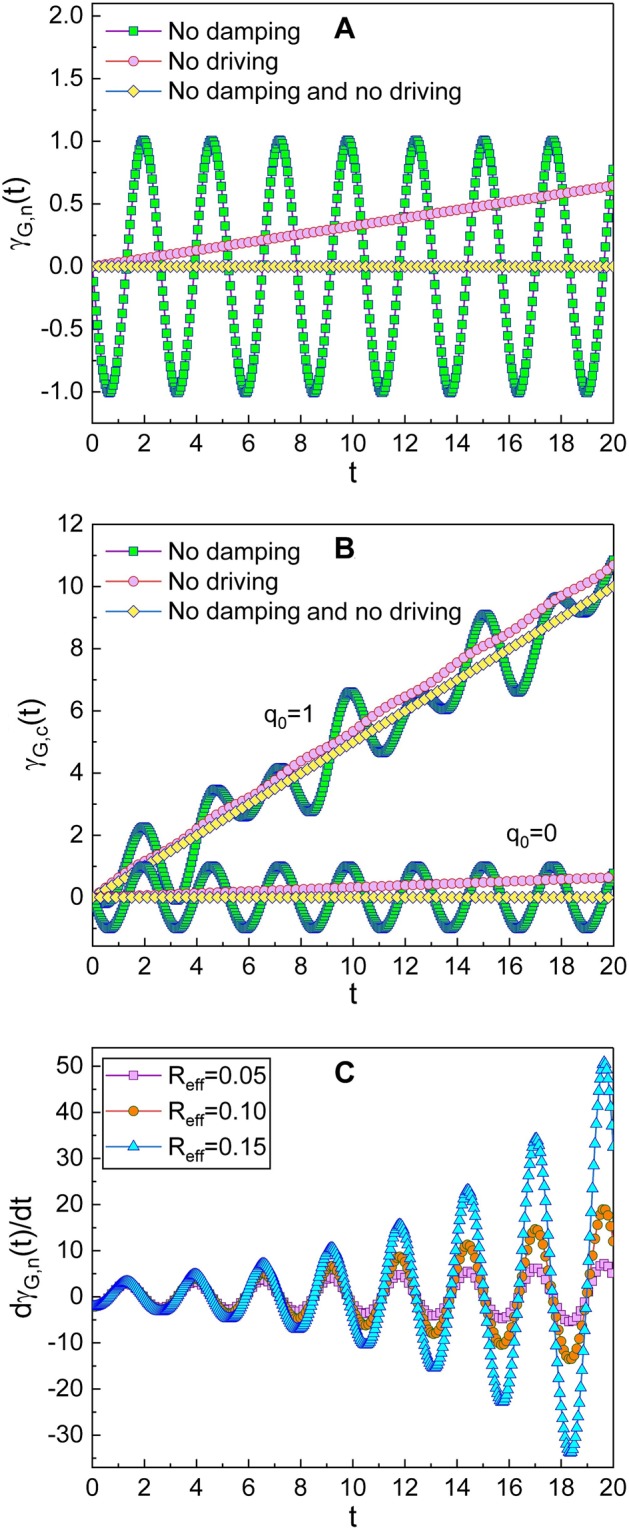
The first two panels are plots of the behavior of the geometric phase for the Fock (**A**) and the coherent (**B**) states under some limitations designated in the legends. For these two panels, the values of (*R*_eff_, *V*_NW_, *V*_eff_) are (0, 2, 1) for the no damping case, (0.5, 0, 0) for the non-driven case, and (0, 0, 0) for the case of no damping together with no driving forces. We have chosen other parameters as *ω* = 2.4, *L*_T_ = 1, *C*_eff_ = 1, *n* = 0, *ℏ* = 1, *γ*_G,*n*_(0) = *γ*_G,*c*_(0) = 0, and *ϕ* = *φ* = 0. The last panel (**C**) is the plot of the time derivative of the geometric phase with the choice of the same values of all parameters as those used in depicting Fig. 1(C).

External driving forces with power sources and the damping of the system may be principal factors responsible for the occurrence of the geometric phase. We can see the influence of the external driving forces on the geometric phase if we remove the damping factor by setting *R*_eff_ = 0 from the graphics. From the graphs involving rectangle boxes filled with green color in Fig. 5(A,B), we can confirm that the external driving forces induce the oscillation of the geometric phase. In the same way, it may be possible to estimate the influence of the damping factor by removing the external driving forces. The graphs plotted together with circles in Fig. 5(A,B) reveal that the non-zero value of the damping constant makes the geometric phase increase with time in a linear manner.

By summing up these, we confirm that, if both the external driving forces and the damping constant are non-zero, the geometric phase oscillates and the value of the upper envelope of such an oscillation increases over time. This leads a significant growth of the oscillation amplitude of the geometric phase as *t* increases.

By the way, for the SHO limit with no driving forces (*V*_NW_ = *V*_eff_ = 0 and *R*_eff_ = 0) in the coherent state, Eq. (36) reduces to

44 $${\gamma }_{G,{\rm{c}}}(t)={\omega }_{0}{\alpha }_{0}^{2}t+{\gamma }_{G,{\rm{c}}}(0).$$

The geometric phase hence increases linearly in this situation. This consequence is shown in Fig. 5(B) (see the graphs joined with rhombuses) and agrees with the result of Ref. 28 (see METHODS).

Because we have considered the geometric phase gained during an arbitrary time *t*^30^ instead of that that corresponds to one-cycle evolution of the state through a closed path, a large increase of the geometric phase even over 2*π* is possible. By the way, if the value of the geometric phase is *γ*_*G*_ = 2*πm* + *ϑ* where *m* is an integer and *ϑ* is an acute angle, we can think that it is actually the same as *ϑ* which is within 2*π*. To examine the rate of the increase of the geometric phase over time, it may be also desirable to see its time derivative. We have shown the time derivative of the geometric phase in Fig. 5(C) as an example. While all chosen values used in depicting Fig. 5(C) correspond to those used in Fig. 1(C) in fact, the graphs reveal a ratio of the geometric phase change, of which scale becomes very large later.

## Conclusion

In this work, the geometric phases of a nanowire-bridged superconducting Fabry-Perot resonator, dominated by a microwave transmitting through it, have been theoretically investigated in both the Fock and coherent states. Through a lumped model of the series RLC circuit, we have described the system in terms of a time-dependent Hamiltonian. Because the traditional method of separation of variables for solving the Schrödinger equation has no merit in this case, we have used other methods, which were the invariant operator method together with the unitary transformation method. Both methods are useful for treating complicated TDHSs. In particular, the unitary transformation of the system with a suitable choice of the unitary operator led to transform the complicated original Hamiltonian to that of the SHO, enabling us to derive the geometric phases thanks to the fact that the mathematical relations associated with the SHO are well known.

Properties of the geometric phases in the system have been analyzed from diverse illustrations. The time behavior of the geometric phase in the coherent state is not so much different from those of the Fock state, while the wave functions for both states are quite different from each other. The geometric phases acquired with the lapse of time more or less oscillate, leading them to exhibiting quasi-periodical time behavior. The amplitude of such oscillations has been increased as time goes by. The influence of the change of parameters, such as *R*_eff_, *C*_eff_, and *q*_0_, on the geometric phases has been analysed.

The main factors that affect on the geometric phase are the external driving forces by batteries together with other power sources and the damping constant. The net effect of the non-zero damping factor on the geometric phase is that it makes the phase increase over time, whereas the external driving forces induce the oscillation of the phase. A synergistic effect stems from combining these two kinds of phase-generating sources is the reason why the geometric phase varies significantly with a high oscillation amplitude later.

In the limit of *V*_NW_ = *V*_eff_ → 0, the geometric phase has been studied in a recent paper of Deymier *et al*. in Ref. 39. While the geometric phase in Ref. 39 was treated within classical mechanics, we have investigated it on the fully quantum basis in this research with strict wave formulations and our results are quite different from theirs. Through this research, we have confirmed that the wave functions accumulate geometric phases through their evolution in time in both the Fock and coherent states. However, in the SHO limit of the system, the geometric phases in the Fock state disappear while the geometric phase in the coherent state does not thoroughly disappear and always remains so long as *q*_0_ is not zero.

This research provides us an understanding to the topological characteristics of the system, that is required to know its exact dynamical properties which are helpful for controlling the operation of the nanowire-based qubits^40^. The knowledge of such theoretical background obtained through our analytical results for phase properties of the system may contribute to promoting the advancement of technologies of nanowire-bridged Fabry-Perot resonators, that are necessary, for example, for their applications on quantum computing systems via qubit fabrications.

## Methods

### Invariant operator method

Because the Hamiltonian is a function of time, it is difficult to solve the Schrödinger equation by means of the conventional separation of variables method. For this reason, we have used another method which is the invariant operator method^32–34^ in order to derive quantum solutions of the system. This method is a part of the principal quantum theory for treating specific dynamical systems that are characterized by time-varying parameters. Because the quantum wave functions of TDHSs are described in terms of the eigenfunctions of the invariant operator^33^, this method is very useful in quantum mechanical treatment of a TDHS. The Liouville-von Neumann equation was used in order to derive the invariant operator of the system. By utilizing the eigenfunctions of the invariant operator, the quantum wave functions, which are necessary for evaluating the geometric phases, are derived.

### Unitary transformation method

The invariant operator that we have introduced in this work is a somewhat complicated form due to the existence of the driving force in the system. In such a case, it is convenient to treat the invariant operator after mathematically transforming it into a simple form by means of the unitary transformation method^34^. Using a unitary operator, the invariant operator has been transformed to that of the SHO whose quantum solutions are well known. By using the unitary relation between the wave functions of the original system and those of the transformed system, we have derived the geometric phases of the system.

### Integral formulae

The time functions *f*_*i*_(*t*) appeared in the text correspond to the following integral formulae, respectively:

45 $${f}_{1}(t)={\int }_{0}^{t}{e}^{\beta t^{\prime} }\,\sin \,\theta (t^{\prime} )\cos \,\theta (t^{\prime} )dt^{\prime} ,$$

46 $${f}_{2}(t)={\int }_{0}^{t}{e}^{\beta t^{\prime} }{\cos }^{2}\theta (t^{\prime} )dt^{\prime} ,$$

47 $${f}_{3}(t)={\int }_{0}^{t}{e}^{\beta t^{\prime} }\,\sin \,\theta (t^{\prime} )dt^{\prime} ,$$

48 $${f}_{4}(t)={\int }_{0}^{t}{e}^{\beta t^{\prime} }\,\cos \,\theta (t^{\prime} )dt^{\prime} ,$$

49 $${f}_{5}(t)={\int }_{0}^{t}{e}^{\beta t^{\prime} \mathrm{/2}}\,\sin \,\theta (t^{\prime} )\cos ({\rm{\Omega }}t^{\prime} +\phi )dt^{\prime} ,$$

50 $${f}_{6}(t)={\int }_{0}^{t}{e}^{\beta t^{\prime} \mathrm{/2}}\,\cos \,\theta (t^{\prime} )\cos ({\rm{\Omega }}t+\phi )dt^{\prime} ,$$

51 $${f}_{7}(t)={\int }_{0}^{t}\,\sin \,\mathrm{[2(}{\rm{\Omega }}t^{\prime} +\phi )]dt^{\prime} ,$$

52 $${f}_{8}(t)={\int }_{0}^{t}\,{\cos }^{2}({\rm{\Omega }}t^{\prime} +\phi )dt^{\prime} ,$$

53 $${f}_{9}(t)={\int }_{0}^{t}{e}^{\beta t^{\prime} /2}\,\sin \,\theta (t^{\prime} )\sin \,({\rm{\Omega }}t^{\prime} +\phi )dt^{\prime} ,$$

where *θ*(*t*) is defined in the text.

### Comparison with other researches

The geometric phase has also been proposed in Eq. (5.13) of Ref. 29 in a limit of our system, where *β* = 0, *V*_NW_ = 0 (but, *V*_eff_ ≠ 0), and *ϕ* = 3*π*/2. This is given by

54 $${\gamma }_{G,n}^{{\rm{L}}{\rm{K}}{\rm{J}}}=2\pi r[|{\beta }_{0}^{2}|+\frac{{V}_{{\rm{e}}{\rm{f}}{\rm{f}}}({\beta }_{0}-{\beta }_{0}^{\ast })\omega }{i\sqrt{2{L}_{{\rm{T}}}{\omega }_{0}}({\omega }_{0}^{2}-{\omega }^{2})}+\frac{{V}_{{\rm{e}}{\rm{f}}{\rm{f}}}^{2}}{2{L}_{{\rm{T}}}{\omega }_{0}}(\frac{{\omega }_{0}^{2}+{\omega }^{2}}{{({\omega }_{0}^{2}-{\omega }^{2})}^{2}}-\frac{1}{{\omega }_{0}^{2}-{\omega }^{2}})],$$

where *r* is a positive integer, and *β*_0_ is an integration constant. In this expression, some notations in their original paper have been changed without loss of generality regarding the notation in the present work.

For the SHO subjected to the coherent state, Biswas and Soni derived the geometric phase accumulated during a one-cycle evolution of the state along a closed path in the parameter space (see Eq. (10) of Ref. 28). It is given by

55 $${\gamma }_{G,{\rm{c}}}^{{\rm{BS}}}=\frac{1}{2}{\oint }_{{\mathscr{C}}}(d{\alpha }_{1}{\alpha }_{2}-{\alpha }_{1}d{\alpha }_{2}),$$

where ${\alpha }_{1}=(\alpha +{\alpha }^{\ast })/\sqrt{2}$ and ${\alpha }_{2}=(\alpha -{\alpha }^{\ast })/(i\sqrt{2})$. The equivalent formula of the geometric-phase shift that corresponds to the state evolution over an arbitrary time *t* can be written as

56 $${\gamma }_{G,{\rm{c}}}^{{\rm{BS}}}=\frac{1}{2}\int (d{\alpha }_{1}{\alpha }_{2}-{\alpha }_{1}d{\alpha }_{2}).$$

A minor evaluation using *α* = *α*_0_exp[−*i*(*ω*_0_*t* + *φ*)] results in

57 $$\begin{array}{rcl}{\gamma }_{G,{\rm{c}}}^{{\rm{BS}}}(t) & = & \frac{1}{2i}\int (\alpha d{\alpha }^{\ast }-{\alpha }^{\ast }d\alpha )\\  & = & {\int }_{0}^{t}{\omega }_{0}{\alpha }_{0}^{2}dt+{\gamma }_{G,{\rm{c}}}^{{\rm{BS}}}\mathrm{(0)}\\  & = & {\omega }_{0}{\alpha }_{0}^{2}t+{\gamma }_{G,{\rm{c}}}^{{\rm{BS}}}\mathrm{(0).}\end{array}$$

This is the same as Eq. (44) in the text.
